# TISSUES 2.0: an integrative web resource on mammalian tissue expression

**DOI:** 10.1093/database/bay003

**Published:** 2018-02-12

**Authors:** Oana Palasca, Alberto Santos, Christian Stolte, Jan Gorodkin, Lars Juhl Jensen

**Affiliations:** 1 Novo Nordisk Foundation Center for Protein Research, Faculty of Health and Medical Sciences, University of Copenhagen, Copenhagen, Denmark; 2 Center for non-coding RNA in Technology and Health, Faculty of Health and Medical Sciences, University of Copenhagen, Copenhagen, Denmark; 3 Department of Veterinary and Animal Sciences, Faculty of Health and Medical Sciences, University of Copenhagen, Copenhagen, Denmark; 4 New York Genome Center, New York, NY, USA

## Abstract

Physiological and molecular similarities between organisms make it possible to translate findings from simpler experimental systems—model organisms—into more complex ones, such as human. This translation facilitates the understanding of biological processes under normal or disease conditions. Researchers aiming to identify the similarities and differences between organisms at the molecular level need resources collecting multi-organism tissue expression data. We have developed a database of gene–tissue associations in human, mouse, rat and pig by integrating multiple sources of evidence: transcriptomics covering all four species and proteomics (human only), manually curated and mined from the scientific literature. Through a scoring scheme, these associations are made comparable across all sources of evidence and across organisms. Furthermore, the scoring produces a confidence score assigned to each of the associations. The TISSUES database (version 2.0) is publicly accessible through a user-friendly web interface and as part of the STRING app for Cytoscape. In addition, we analyzed the agreement between datasets, across and within organisms, and identified that the agreement is mainly affected by the quality of the datasets rather than by the technologies used or organisms compared.

**Database URL**: http://tissues.jensenlab.org/

## Introduction

Model organisms have helped understanding many essential biological processes and the genes involved in their normal and abnormal functioning, i.e. in disease. Experiments in model organisms, especially in animal models, have been interpreted and subsequently translated into the human system with great success (1–3). Physiological and molecular similarities across species allow translation from simpler systems to more complex ones. Analysis of the common tissue-expression profiles between animal models and human has been essential to bridge between *in vivo* experiments and translational medicine, which is notably relevant when evaluating toxicological consequences to treatment prior to trials in humans (4–6). Systematic investigation of the physiological and molecular similarities is currently possible in view of the accumulating experimental data on tissue-level expression for different organisms (7–9).

The most widely used techniques for measuring mRNA expression levels on a high-throughput scale are high-density oligonucleotide microarrays and in particular RNA sequencing (RNA-seq). Tissue expression datasets provide data of varying quality, and the technology platform of choice can have an important impact on quality. Provided sufficient sequencing depth, RNA-seq has advantages over microarrays in terms of better discrimination between isoforms and the ability to discover new transcripts (10). Nonetheless, several studies (10–13) have shown that expression estimates derived from RNA-seq and microarray correlate well and complement each other. The quality of datasets can be furthermore influenced by the quality of the genome assembly and annotation of the respective species. Genome quality will have impact on the mapping rate and accuracy in RNA-seq experiments, and on the design of correct oligonucleotides in microarray experiments, while a poor genome annotation will obviously decrease the size of the gene set being measured in each experiment, irrespective whether RNA-seq or microarray. Due to being more studied, human and mouse have better quality genomes than pig, which also has a smaller and less reliable set of annotated genes (14).

Several resources provide spatial information on gene expression based on a single organism (15–17) or a single technology (18), or collecting data on multiples species and technologies (19, 20). Here, we present a database of gene–tissue associations in human and three mammalian model organisms. By gene–tissue association, we denote the expression or simply presence of the mRNA or corresponding protein in that tissue. Certain applications (e.g. cellular signaling or protein interaction pathways) require information about where proteins are present, irrespective of their origin of expression.

The first version of the database was implemented as part of a comprehensive comparison of human tissue expression datasets (21) and integrated multiple datasets made with several technologies. In this version, we further include 10 transcriptomic datasets from mouse, rat and pig including both microarray and RNA-seq derived data for each organism. Most of the RNA-seq data was processed in-house, mapping reads to newest genome assemblies and quantifying transcript levels using latest genome annotations. We integrate this data with tissue expression evidence from manually curated UniProtKB (22) annotations and automatic text mining. All gene–tissue associations provided are benchmarked and scored to make all the data comparable within each organism and between organisms. With such a unified scoring it is possible to compare the associations across organisms and technologies and therefore establish the overall independent confidence of the interactions. Including animal data in TISSUES and making them comparable to the already existing human datasets conspicuously complements the database and allows translation of tissue–expression profiles from any of the species to any of other. All data are freely available via a web interface (http://tissues.jensenlab.org/) where gene tissue expression can be visualized and downloaded, or further through the Cytoscape App, stringApp (http://apps.cytoscape.org/apps/stringapp), which allows users to easily visualize tissue expression into a network from the STRING database.

## Materials and methods

In this new version of the TISSUES database, we add tissue expression data from mouse, rat and pig to the existing human gene–tissue associations. In this effort, we integrate data collected from different sources of evidence (transcriptomics, text mining and manual curation) and make them comparable both among datasets and across species. For the transcriptomics datasets, we chose large-scale experiments with expression measured in several tissues in healthy individuals. We have specifically selected samples corresponding to the adult life stage in all datasets except from one of the pig datasets, where all the animals used in the study were juvenile (12–16 weeks old).

As described below, we score each of the datasets according to their quality by benchmarking them against a gold standard set of gene–tissue associations derived from the UniProtKB tissue annotations for human proteins. This common scoring scheme facilitates correlation analyses between datasets, between the species covered, and, at the gene level, for homologs.

A first step in integrating tissue expression data from multiple sources is standardizing gene and tissue identifiers. We obtained a dictionary of aliases (gene/protein names) and identifiers for protein-coding genes for all four organisms from the STRING database (23). We used this dictionary to map the gene/transcript identifiers in each dataset to the corresponding Ensembl protein identifiers (from now referred to as STRING identifiers). When two or more genes/transcripts mapped to the same STRING identifier, we used the average of their expression value. If a gene/transcript could be mapped to more than one STRING identifier, it was filtered out.

To standardize the names used for tissues, we mapped all tssues available in the datasets to the Brenda Tissue Ontology (BTO) (24). The directed acyclic graph structure of ontologies allowed us to propagate expression calls to parent tissues. We selected a set of 21 major tissues for assessing the agreement between datasets and for visualization in the web interface. The mapping from tissue names to BTO terms is available at doi:10.6084/m9.figshare.4640710.

### Text mining

We used the dictionaries together with a previously published and highly efficient named entity recognition (NER) engine (25) to identify, using dictionaries, genes/proteins and tissues co-mentioned in Medline abstracts (26). Species disambiguation of names was performed by looking for explicit mentions of the species names, including both Linnaean binomina and common names, as in the STRING database (23).

The NER results were used to extract scored gene–tissue associations for the studied species. The co-occurrence of gene–tissue terms is scored based on whether the terms appear within the same sentence or only within the same abstract (weighted counts) normalized by how much the gene and tissue is mentioned with other tissues/genes (27). The final result was a set of gene–tissue associations for mouse, rat, and pig genes, which had been extracted and scored exactly as previously described for human genes (21).

### Mouse transcriptomics datasets

#### Mouse GNF Gene Expression Atlas

We downloaded the microarray data from the Mouse GNF1M Gene Atlas (GSE1133) (28), measuring expression in C57BI/6 mice, from the BioGPS portal (18). The dataset contains information for 36 182 probe sets (including controls), which we first mapped to gene identifiers via the probeset annotation file (gnf1m.annot2007.tsv) and then to STRING identifiers via our dictionary. Expression values were averaged across probesets corresponding to the same gene and across biological replicates. The mapped version of the GNF Mouse Gene Expression Atlas from BioGPS provides information on 15 390 genes in 78 tissues/cell types, 64 of which could be mapped to BTO terms.

#### Mouse GNF V3 Gene Expression Atlas

The microarray data from the GNF Mouse GeneAtlas V3 (GSE10246) (29), with expression measured in C57BI6 mice, was also obtained from BioGPS. The data were already normalized and expression values were averaged across bio-replicates. We downloaded a probeset annotation file corresponding to the MOE430_2 array platform from BioMart (30) and used it for mapping probe identifiers to Ensembl gene identifiers. These were further mapped to 16 795 STRING identifiers using our dictionary, and 64 out of the total of 91 tissues and cell types were mapped to BTO terms.

#### Mouse RNA-seq for evolutionary dynamics, MIT

In this atlas, which also covers four other mammals including rat, RNA-seq was used to sequence polyA-selected RNA from nine tissues in mouse with three biological replicates (31). Two of the three biological replicates were inbred strains, while the third was from an outbred line. Paired-end sequencing was performed with fragments of 36–50 bases in two individuals and, respectively, 80 bases in the third individual. We downloaded the FASTQ files from ArrayExpress (32) (E-MTAB-2801) and processed it using the pipeline described later. We quantified expression in FPKM units for 22 048 STRING identifiers and mapped all nine tissues to BTO terms.

#### Mouse RNA-seq, ENCODE/CSHL

The mouse transcriptomic data produced at CSHL under the ENCODE project (33) was similarly based on RNA-seq to sequence polyA-selected RNA, in this case generating paired-end Illumina data (2× 101 bases) for 22 tissues with two biological replicates in C57BI/6 mice (34). We downloaded the files containing gene expression quantification in FPKM units for each sample from the Encode Portal (35). We were able to map 21 of the 22 tissues to BTO terms and a total of 22 048 STRING protein identifiers.

### Rat transcriptomics datasets

#### Rat Array Atlas

The expression of ∼7000 rat genes was measured by array across 11 peripheral and 15 brain tissues in Sprague Dawley, Wistar and Wistar Kyoto rats, using two to three samples from different or same strain per each tissue (36). Normalized data were obtained via BioGPS. The annotation file for the RG_U34A array chip used in the study was used for mapping the 7999 probeset identifiers to a total number of only 4731 STRING identifiers. 25 of the 26 tissue names could be mapped to BTO terms.

#### Rat transcriptomic BodyMap

The Rat transcriptomic BodyMap study used RNA-seq to profile expression across 11 tissues and four developmental stages for 32 individuals in Fisher 344 rats (37). We obtained processed data for the adult developmental stage with expression measured in FPKM units and annotated with Ensembl gene identifiers from ArrayExpress (E-GEOD-53960). We further mapped these to 19 554 STRING identifiers using the dictionary and averaged the expression values across biological replicates.

#### Rat RNA-seq for evolutionary dynamics, MIT

This data come from the same study as the previously described MIT RNA-seq mouse atlas (31). Expression was measured using RNA-seq on polyA-selected RNA from the same nine tissues with three biological replicates. We downloaded the FASTQ files from ArrayExpress (E-MTAB-2800) and processed them using our pipeline, thereby quantifying expression for 19 566 STRING identifiers.

### Pig transcriptomics datasets

#### Pig Array Atlas

The study used microarrays to measure expression in 62 tissues/cell types in juvenile Landrace pigs, including genes inferred by orthology and not annotated in the pig genome at the time (38). From the BioGPS portal we obtained the RMSD normalized expression values for probesets, which we mapped to gene names via the annotation provided in the file and further to STRING identifiers via our dictionary. For each of the resulting 14 850 protein-coding genes, we averaged expression across probesets and biological replicates. We were able to map 53 of the tissues/cell types to BTO terms.

#### Pig RNA-seq Aarhus

This dataset consists of RNA-seq data on polyA-selected RNA from ten porcine tissues with two biological replicates in Landrace boars (39). Since only BAM files with TopHat alignments against the Sus_scrofa.Sscrofa10.2 genome build were available, we downloaded these from ArrayExpress (E-MTAB-1405) and performed transcript abundance quantification and normalization across all samples using Cuffnorm from the Cufflinks suite (40).

#### Pig RNA-seq WUR

The dataset produced at the Wageningen University under the FAANG project (Functional Annotation of Animal Genomes) consists of RNA sequencing data for eight porcine tissues and three different breeds (duroc, large white, pietrain). Five of the tissues were sequenced in one individual from each breed, whereas the remaining three tissues were sequenced only in one individual (large white). We obtained the raw FASTQ files from the European Nucleotide Archive (study: PRJEB19268, ERP021264) and processed the data using our RNA-seq processing pipeline. We treated the samples from different species as biological replicates, averaging the expression values for each gene across the three individuals when available.

### Human transcriptomics datasets

To assess how well datasets agree with each other across organisms, we further make use of the four human transcriptomic datasets, which were already integrated and described in detail in the first version of TISSUES (21). Two of them were obtained by microarray technologies, namely Exon Array (41) and GNF (28), and the other two by RNA-seq, namely HPA RNA-seq (42) and RNA-seq Atlas (43).

### Processing of RNA-seq datasets

For mapping raw RNA-seq reads to reference genomes and quantifying gene expression levels, we used the genome assemblies (mmgrc38ens83, rnor6ens83 and Sus_scrofa.Sscrofa10.2) and annotation files (Mus_musculus.GRCm38, Rattus_norvegicus.Rnor_6.0 and Sus_scrofa.Sscrofa10.2) corresponding to Ensembl release 83 (44).

We used the STAR RNA-seq aligner (45), version 2.5.0b, to map reads to reference genomes. In order to keep higher confidence alignments only, we filtered out alignments containing non-canonical splice junctions (–outFilterIntronMotifs RemoveNoncanonical), as well as novel splice sites of low confidence (–outFilterType BySJout).

To quantify expression and normalize expression values across samples, we ran Cuffnorm v2.2.1 (40) directly on the BAM files generated by STAR, together with gtf annotation files. The RNA-seq processing pipeline can be found at https://github.com//opalasca/rnaseq_processing.

### Gold standard

#### Manually curated annotation

To evaluate the quality of the gene–tissue associations from each dataset and to assign directly comparable confidence scores, we compare all datasets to a gold standard as was done in the first version of TISSUES (21). We import the manual annotations of tissue expression provided by UniProtKB for all four organisms (46), which we include in the Knowledge channel of the database.

Because the number of manual tissue annotations in mouse, rat, and especially pig is much lower than in human (Supplementary Table S1), we cannot directly use them as gold standard for deriving confidence scores. Instead, we opted to generate gold standard datasets for mouse, rat, and pig through orthology-based transfer of the human tissue annotations, under the assumption that a large portion of orthologous genes are similarly expressed in homologous tissues across mammals (31,47).

#### Orthology-based transfer of annotations

Ortholog/paralog assignments were derived from the orthologous groups (OGs) defined in eggNOG 4.5 (48). Specifically, we downloaded the files comprising OGs across mammals (maNOG.members.tsv.gz) and across rodents (roNOG.members.tsv.gz) from the eggNOG download page. We used the latter file to obtain 1:1 orthologs between mouse and rat, and the former file for all other orthology relationships. Because eggNOG and STRING use the same identifiers, no identifier mapping was necessary.

To construct the gold standard dataset in each of the three animal models, we used only 1:1 orthologs between human and the organism in question. For the comparative analyses of datasets across technologies and organisms, we extracted both 1:1 orthologs between each pair of organisms and 1:1:1:1 orthologs across all four organisms (Supplementary Tables S2 and S3).

### Confidence scoring

**Figure 1.**
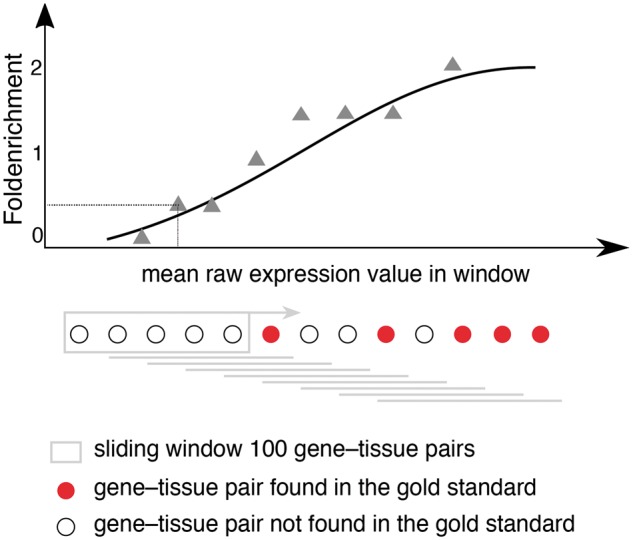
Schematic representation for calculating fold enrichment and fitting its relationship to raw expression values. For a given dataset, we select gene–tissue pairs and their raw expression scores for a subset of tissues, which are common between all datasets and a subset of genes common between the dataset and the gold standard. Next, we sort these gene–tissue pairs by their raw expression value, and traverse them in sliding windows of a pre-defined size. The enrichment corresponding to each bin is then calculated as the fraction of gene–tissue pairs from that bin found in the gold standard, divided by the fraction of pairs that would be expected by random. Next, we use appropriate functions (see text) to fit the relationship between fold enrichment values and mean raw expression values in their corresponding windows.

To make the datasets comparable, we converted the raw expression scores into a common scoring scheme. This scheme requires first evaluating the agreement of the datasets with the gold standard for the corresponding organism. As in the previous TISSUES version (21), we quantify this agreement using fold enrichment and convert raw expression into confidence scores. Fold enrichment is defined as the fraction of gene–tissue pairs in the dataset also found in the gold standard divided by the fraction expected when randomly sampling genes and tissues from the gold standard. To calculate the fold enrichment in a given dataset, we select the genes and tissues that are in common between the dataset and the gold standard, sort the gene–tissue pairs by raw expression value and calculate fold enrichment in sliding windows of 100 pairs (Figure 1). We then fit the resulting curves (raw expression, fold enrichment) using sigmoidal functions to define how raw expression scores translate into fold enrichment (confidence score) for each dataset:

$$Confidence\;score=a_{0}+\left(a_{1}-a_{0}\right)/\left(1+e^{{-a}_{2}\left({log}_{10}\left(x\right)-a_{3}\right)}\right),$$

where x is the mean expression value of a 100-pairs bin.

To test if the use of orthology-based inference in the gold standard affected the fold enrichments, we repeated these analyses using instead the actual UniProtKB tissue annotations for each organism as gold standard. These analyses gave consistent but less robust results due to the use of much smaller gold standard sets, in particular for rat and pig (Supplementary Figure S1). Since the number of protein–tissue pairs in the orthology-based gold standard sets are almost 2-fold smaller than the original human gold standard, we also tested whether the previously published results (21) for transcriptomic datasets were reproducible with a gold standard reduced to 1:1 orthologs with either of the three organisms. We observed only minor changes in fold enrichment values, indicating that the scores are robust, irrespective of size of the gold standard dataset (Supplementary Figure S2).

The final step in our scoring scheme is to transform the confidence scores into stars, which are used to score other types of evidence than expression datasets within the TISSUES database. Stars represent the confidence that a gene is expressed or present at all in a certain tissue. Gene-tissue associations with negative scores are not included in the database. To this end, we used a single, monotonic calibration function for all datasets from all organisms, which we calibrated on the text-mining results for human gene–tissue associations (Supplementary Figure S3). We chose to use this particular set of associations, because it is large, facilitating robust results, and because text-mining is also available for other types of associations, allowing unified confidence scores across TISSUES and the related databases COMPARTMENTS (27) and DISEASES (49). The calibrated functions used for transforming raw expression values into final confidence star scores are available in Supplementary Table S4.

### Correlation between datasets

We analyzed the agreement between datasets across organisms and technologies (microarray vs. RNA-seq) by computing Pearson correlation coefficients between the final star confidence scores for all pairs of datasets. We performed computations both at the level of individual tissues and across multiple tissues, the latter by pooling gene–tissue associations from the tissues that are common between each pair of datasets. To calculate the correlation between two datasets, we constructed vectors of the final star confidence scores for gene–tissue pairs involving only genes observed in both datasets. When comparing datasets from different organisms, 1:1 orthologs were used for selecting the set of common observed genes. Associations with negative scores (stars) were filtered out prior to this analysis.

## Results and discussion

**Figure 2.**
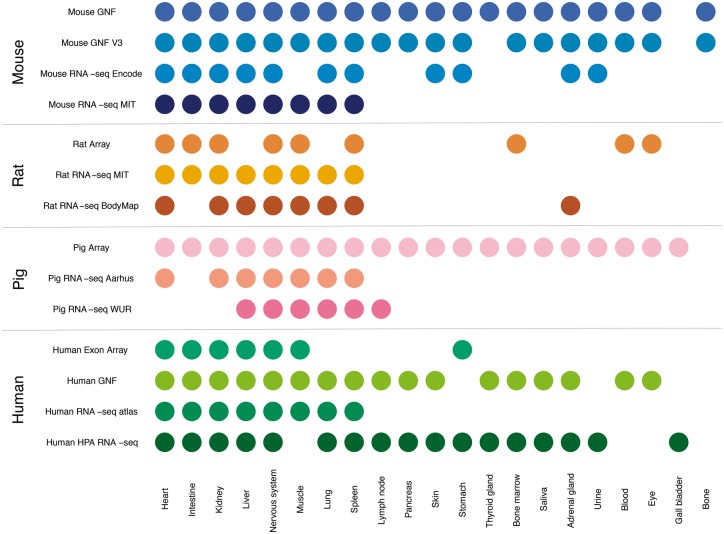
Summary of tissues present in each dataset. We mapped the newly integrated datasets from mouse, rat and pig, as well as the already existing human datasets, to 21 major tissues of interest. This figure shows which of these tissues are covered by which datasets.

In this study, we compare gene expression in human and three mammalian model organisms across 14 different transcriptomic datasets and 21 major tissues. Figure 2 shows an overview of which tissues are covered by which datasets. We present results from benchmarking of all the datasets and quantify the agreement between tissue expression data created using different organisms and different transcriptomic technologies (RNA-seq vs. microarrays). Finally, we describe a web resource that makes it easy to view the tissue expression of a gene of interest.

### Benchmarking of model organisms

#### Correlation between raw expression values and fold enrichment

**Figure 3.**
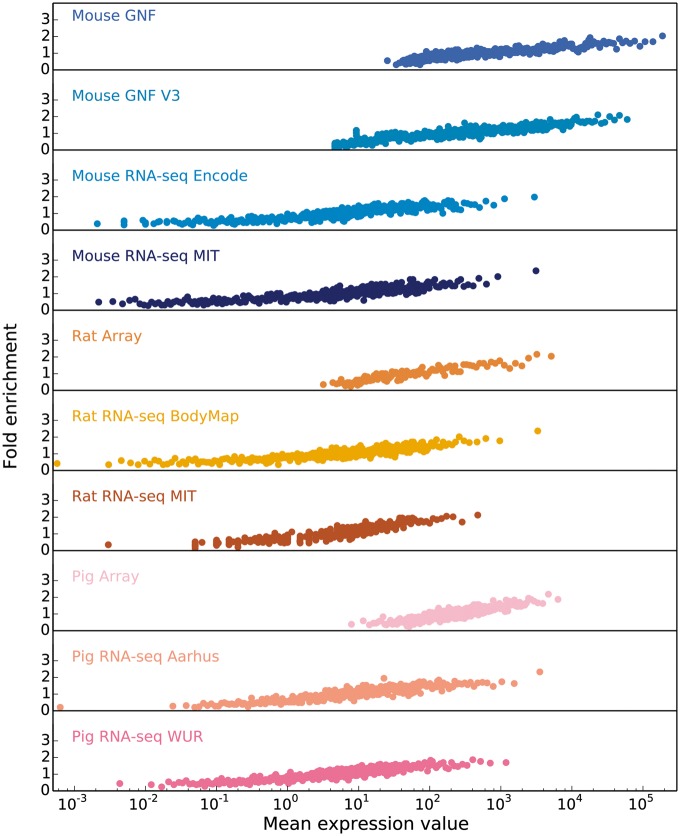
In order to obtain confidence scores for each gene–tissue pair, we assess the relationship between raw expression values and fold enrichment, defined as the agreement between each dataset and gold standard datasets specific to the organism. The gold standard datasets are based on the UniProtKB protein tissue annotations in human, filtered for 1-to-1 orthologs between human and each of the three organisms. The *x*-axis contains raw expression values for gene–tissue pairs, in units specific to the type of experiment or processing of data (e.g. intensity units for microarray studies, FPKMs for RNA-seq), and averaged across bins of 100 pairs.

Starting from the intuition that the higher the estimated abundance level for a specific gene in a tissue, the more confident we are that the gene is expressed in that tissue, we assess the correlation between expression values and confidence by comparing each transcriptomic dataset from each organism to a gold standard dataset corresponding to that organism. Since the gold standard datasets, derived from UniProtKB gene–tissue associations for human, are reliable but very incomplete, we cannot estimate the precision of a dataset. Instead, the quality of each dataset is estimated in terms of the fold enrichment of gene–tissue associations found within the gold standard compared to random chance. As expected, the comparison showed a clear correlation between fold enrichment and raw expression values (Figure 3).

### Comparison of expression across organisms

To assess how well expression from a model organism can be used as a proxy for expression in human, we analyzed how well the datasets agreed with each other across organisms and across technologies (microarray vs. RNA-seq). For this, we performed two distinct analyses. First, we computed Pearson’s correlation coefficients for the vectors of confidence scores of gene–tissue associations across all pairs of datasets. Second, we benchmarked all the datasets against the UniProtKB gold standard to show how quality of the datasets influences the measured agreement.

**Figure 4.**
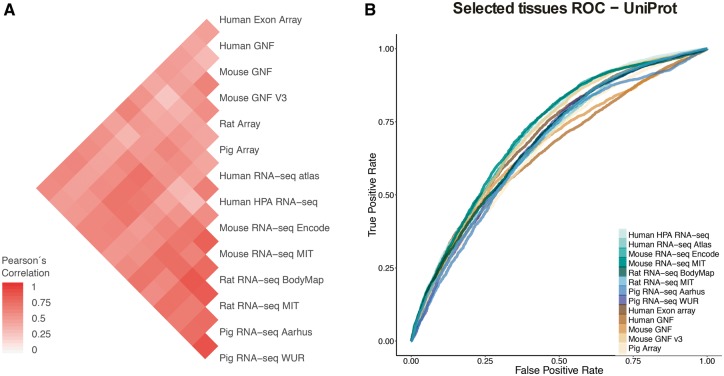
(**A**) Pearson’s correlation coefficients between final confidence scores of gene–tissue associations across datasets. For each pair of datasets, we considered the set of common genes (genes being expressed in at least one tissue in each dataset), and common tissues between the two datasets. (**B**) In this panel, we compare the rates of True positives and False positives for each dataset for the common tissues to show that the correlation between datasets is mainly influenced by quality rather than by organism or technology.

In the correlation analyses, we first computed the correlation between each two datasets based on the set of common tissues and common genes, using 1:1 orthology relationships when datasets belonged to different organisms (Figure 4A). To account for possible biases arising from this, we repeated the analysis for individual tissues, using the set of genes common to all datasets (Supplementary Figure S4). We additionally looked at the correlation across tissues and datasets for six tissues covered by most of the datasets, which clearly showed that tissues cluster together across datasets irrespective of the organism of origin (Supplementary Figure S5).

Second, to study the possible causes of the observed agreement or disagreement between datasets, we looked at the Receiver Operating Characteristic curves (ROC curves) resulting from the comparison of each dataset to gene–tissue pairs in UniProtKB. The ROC curves account for the ratio of true positives and false positives as a function of the calibrated scores (Figure 4B). The True Positive Rate (TPR) and False Positive Rate (FPR) are calculated taking into account only genes shared between UniProtKB and each of the benchmarked datasets and four common tissues to most of these datasets (nervous system, liver, heart and kidney). RNA-seq WUR dataset was evaluated using only nervous system and liver tissues since the other two tissues were not available.

Both analyses show that the agreement between datasets is mainly driven by the technical bias in the data as well as the difference in quality of the datasets. We do not observe a clustering of the datasets by either organism or technology (microarray vs. RNA-seq). However, we observe the strongest correlations between datasets produced in the same lab, i.e. mouse MIT with rat MIT and mouse GNF with mouse GNF v3, indicating that the technical bias plays an important role in how well datasets agree with each other. We also observe that certain datasets have an overall higher agreement to all the other datasets (HPA RNA-seq or mouse/rat MIT). We interpret this as a result of their overall quality, the higher the quality of the dataset the better the agreement with other datasets. Indeed, the datasets that correlate well with other datasets (i.e Human HPA RNA-seq) show also better performance in the ROC curves than other datasets.

### The TISSUES web resource

The TISSUES database can be accessed via the web interface (http://tissues.jensenlab.org), which is designed to cater to users who want to inspect the tissue–expression of a protein of interest. The user is first presented with a search interface that retrieves proteins by name, using the synonyms list also used for the text mining evidence channel. Upon selecting a protein, the user is presented with a results page, which contains an anatomical schematic and three tables with further details on the expression evidence.

**Figure 5.**
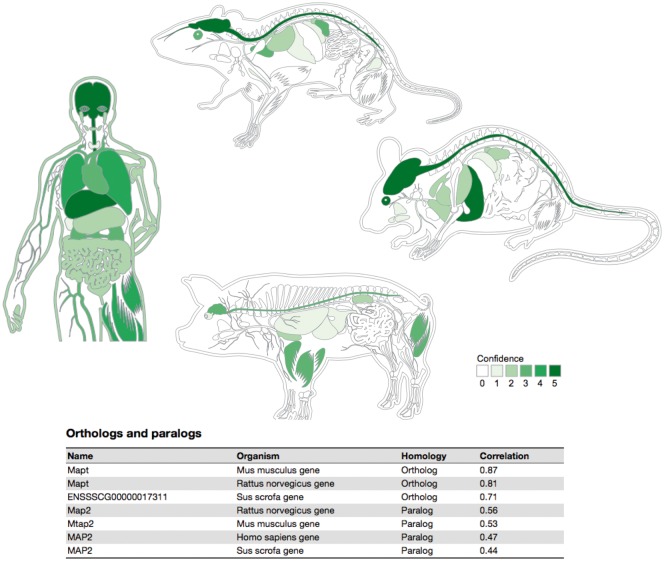
Summary figures for all the covered organisms. The web interface provides a comprehensive figure for each organism where the tissue associations for the queried gene are summarized. In this example, we are showing the tissue expression profile for the Microtubule-associated protein tau (MAPT) known to be related to Alzheimer’s disease (57). The ortholog–paralog table provides information about homologous proteins and their tissue–expression correlation with the query protein.

To provide a high-level overview of the tissue expression of a protein, we created an anatomical schematic for each of the four organisms (Figure 5). The schematics cover 20 major tissues for the rat and 21 for the three other organisms, the difference being that rats do not have a gall bladder (50). For a given protein, each tissue is colored based on the integrated confidence score, which takes into account all evidence types. Hovering over a tissue causes a popup to be shown, which summarizes the sources of evidence that support expression of the protein in the tissue.

Whereas the anatomical schematic gives an overview of tissue expression, it does not provide the full details of the evidence in TISSUES. For this reason, the results page also contains three tables, one for each of the three evidence channels. These tables list the precise ontology terms for the site of expression, thus often providing more fine-grained information than the schematic. The Knowledge and Experiments table lists the source of each piece of evidence and contains a link out to the original source when possible. In case of the Text mining table, we instead provide an evidence viewer, which displays the abstracts that co-mention a protein and a tissue, highlighting the recognized terms in the text.

To provide evolutionary context, a last table on the page summarizes the tissue expression of orthologs and paralogs of the query protein. In this table, homolog proteins are annotated as either orthologous or paralogous proteins according to EggNOG database (48). Each of the entries in this table shows the tissue–expression correlation with the query protein for the 21 major tissues (Pearson’s correlation coefficient), which facilitates comparison between organisms.

A separate download page provides links to tab-delimited files with the complete data for each evidence channel as well as a tab-delimited file with the integrated confidence score for every protein–tissue association. These files are made available under the Creative Commons Attribution 4.0 license to facilitate both large-scale analyses and incorporation of the data into other databases. Examples of the latter include Hetionet (https://github.com/dhimmel/hetionet) and Pharos (51).

### Integration with Cytoscape and STRING

Tissue expression is not just relevant for understanding the roles of individual proteins; it is also important for analysis of protein–protein interaction networks (52). Recently, there has been a growing interest in developing resources that combine data on tissue expression and protein interaction networks (53, 54). The TISSUES database itself does not include interaction data; however, the synchronization of protein identifiers with the STRING database enables easy integration of the two (23, 55).

**Figure 6.**
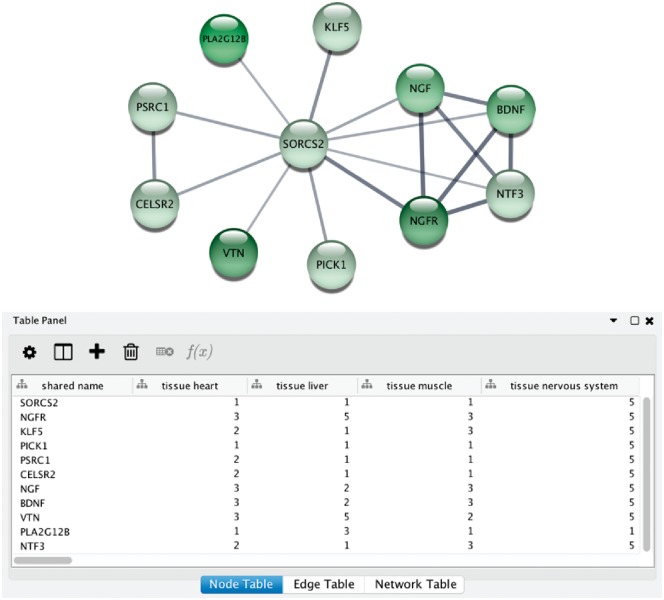
STRING app in Cytoscape with tissue information. (**A**) The newly developed stringApp for Cytoscape allows users to get all the STRING functionality within Cytoscape and allows expression evidence from TISSUES to be visualized onto the network (in this example for liver). (**B**) The stringApp also shows the evidence score for each of the major tissues in the node attributes table.

Integrative analysis of protein networks and tissue expression data from STRING and TISSUES, respectively, has recently been greatly simplified with a new STRING app for the Cytoscape software platform (56) (http://apps.cytoscape.org/apps/stringapp). When using the app to retrieve a STRING network for one of the organisms covered by TISSUES, the combined evidence score for each of the major tissues is automatically included as node attributes. This allows them to be directly used for subsequent filtering of the network or to be mapped onto the network, as exemplified in Figure 6. Full details and a guide on how to use the STRING app will be described in a separate publication.

The code used to obtain fold enrichment scores and to generate the figures in the manuscript can be downloaded at https://github.com/opalasca/TISSUES_Update.

## Conclusions

We presented TISSUES 2.0, an updated database on gene–tissue associations that has now been substantially extended by including information from multiple mammalian model organism, namely mouse, rat and pig. This database is an integrative effort that provides tissue associations protein-coding genes, collected from multiple sources and data types. The associations have been benchmarked and assigned confidence scores to make them comparable across data sources, data types, and organisms. The database further facilitates comparisons across organisms by showing gene–tissue correlations between orthologous and paralogous genes.

TISSUES 2.0 integrates both transcriptomics and proteomics data as well as manually curated and automatically text-mined associations from the biomedical literature. It therefore provides information of where proteins are present in the body, rather than only where they are expressed. This distinction is particularly important for signaling proteins, such as insulin, that are produced in one tissue but transported elsewhere through the blood. Thus, TISSUES 2.0 is complementary to resources purely based on transcriptomics data. The resource is already integrated into both GeneCards and BioGPS (via a plugin).

The database is available through a web interface that allows the data to be queried, visualized and compared in a gene-centric manner across data sources and organisms. The web resource also makes all gene-tissue associations freely available for downloaded. Furthermore, TISSUES 2.0 is now accessible through the Cytoscape STRING app, thereby providing biological context to the STRING protein–protein interaction networks by adding tissue information.

## Supplementary data

Supplementary data are available at *Database* Online.

## Supplementary Material

Supplementary Data
