# TISSUES 2.0: an integrative web resource on mammalian tissue expression

**DOI:** 10.1093/database/bay028

**Published:** 2018-03-16

**Authors:** Oana Palasca, Alberto Santos, Christian Stolte, Jan Gorodkin, Lars Juhl Jensen

*Database* (2018), doi: 10.1093/database/bay003

In the original article, the wrong author name was indicated next to the ‘Correspondence may also be addressed to’ section. It should have read as follows:

*Corresponding author: Tel +45 35325025; Email: lars.juhl.jensen@cpr.ku.dk

Correspondence may also be addressed to Jan Gorodkin. Tel: +45 35333578; Email: gorodkin@rth.dk

This has now been corrected online. The Publisher apologizes for the error.

