# Supplementary material for: TISSUES 2.0: an integrative web resource on mammalian tissue expression
Source: Database (Oxford). 2018 Feb 12;2018:bay003. doi: 10.1093/database/bay003 (PMC5808782; doi:10.1093/database/bay003)
Supplement: Supplementary Data [file bay003_supp.docx]

Supplementary Material

TISSUES 2.0: An integrative web resource on mammalian tissue expression

Oana Palasca^1,2,3^*, Alberto Santos^1^*, Christian Stolte^4^, Jan Gorodkin^2,3#^, Lars Juhl Jensen^1,2#^

^1^ Novo Nordisk Foundation Center for Protein Research, Faculty of Health and Medical Sciences, University of Copenhagen, Denmark
^2^ Center for non-coding RNA in Technology and Health, Faculty of Health and Medical Sciences, University of Copenhagen, Denmark
^3^ Department of Veterinary and Animal Sciences, Faculty of Health and Medical Sciences,University of Copenhagen, Denmark
^4^ New York Genome Center, United States
* These authors contributed equally.
^#^ Corresponding authors: LJJ : [lars.juhl.jensen@cpr.ku.dk](mailto:lars.juhl.jensen@cpr.ku.dk); JG: [gorodkin@rth.dk](mailto:gorodkin@rth.dk)

|  | Proteins | BTO terms | Protein-tissue pairs |
| --- | --- | --- | --- |
| Human | 16,645 | 399 | 64,469 |
| Mouse | 10,760 | 177 | 28,476 |
| Rat | 4,605 | 151 | 5,551 |
| Pig | 617 | 93 | 729 |

*Supplementary Table S1. Size of the UniProtKB (release august 2017) tissue annotation datasets in each organism.*

| Organisms | 1:1 orthologs |
| --- | --- |
| Human – Mouse | 12,736 |
| Human – Rat | 11,038 |
| Human – Pig | 10,916 |
| Mouse – Rat | 12,959 |
| Human – Mouse – Rat – Pig | 8,665 |

*Supplementary Table S2. Size of the 1:1 orthologous protein sets obtained from eggNOG 4.5. OGs across rodents were used for extracting the mouse-rat orthologs, and OGs across mammals were used for extracting the other orthology relationships.*

|  | Proteins | BTO terms | Protein-tissue pairs |
| --- | --- | --- | --- |
| Mouse | 11,530 | 381 | 46,464 |
| Rat | 9,972 | 375 | 40,038 |
| Pig | 9,922 | 370 | 40,233 |

*Supplementary Table S3. Size of the gold standard datasets in mouse, rat and pig, derived from the UniProtKB tissue annotation in human, by transferring annotations to their 1:1 orthologs in each of the other organisms.*

| Dataset | Confidence score (stars) |
| --- | --- |
| Mouse GNF | $5.29/\left( 1+{7.84 x}^{-0.34} \right)-2$ |
| Mouse GNF V3 | $5.44/\left( 1+{6.79 x}^{-0.31} \right)-1.65$ |
| Mouse RNA-seq ENCODE | $3.56/\left( 1+{3.77 x}^{-0.7} \right)-0.96$ |
| Mouse RNA-seq MIT | $4.46/\left( 1+{3.36 x}^{-0.38} \right)-0.88$ |
| Rat Array | $5.16/\left( 1+{7.97 x}^{-0.47} \right)-$1.63 |
| Rat RNA-seq MIT | $5.78/\left( 1+{5.35 x}^{-0.27} \right)-0.61$ |
| Rat RNA-seq BodyMap | $4.3/\left( 1+{5.4 x}^{-0.55} \right)-0.55$ |
| Pig Array | $4.98/\left( 1+{29.79x}^{-0.58} \right)-1.40$ |
| Pig RNA-seq Aarhus | $4.56/\left( 1+{2.60 x}^{-0.48} \right)-1.53$ |
| Pig RNA-seq WUR | $4.28/\left( 1+{2.51 x}^{-0.44} \right)-1.14$ |

*Supplementary Table S4. Calibrated functions used for transforming expression scores into final star confidence scores, where* $\left. x \right.$ *represents the raw expression value. The functions listed in this table combine the sigmoidal functions translating raw expression values into fold enrichment scores, with the monotonic functions transforming fold enrichment scores into final star confidence scores. The parameters for the sigmoidal functions are obtained using the fit_curves function implemented in the scipy.optimize Python package.*

*Supplementary Figure S1. Comparison between using UniProtKB tissue annotations in each organism as gold standard datasets (right) vs. deriving the gold standard based on 1:1 orthology to human in each of the three organisms (left). We observe consistent results in all three organisms, but less robust in rat and especially in pig.*


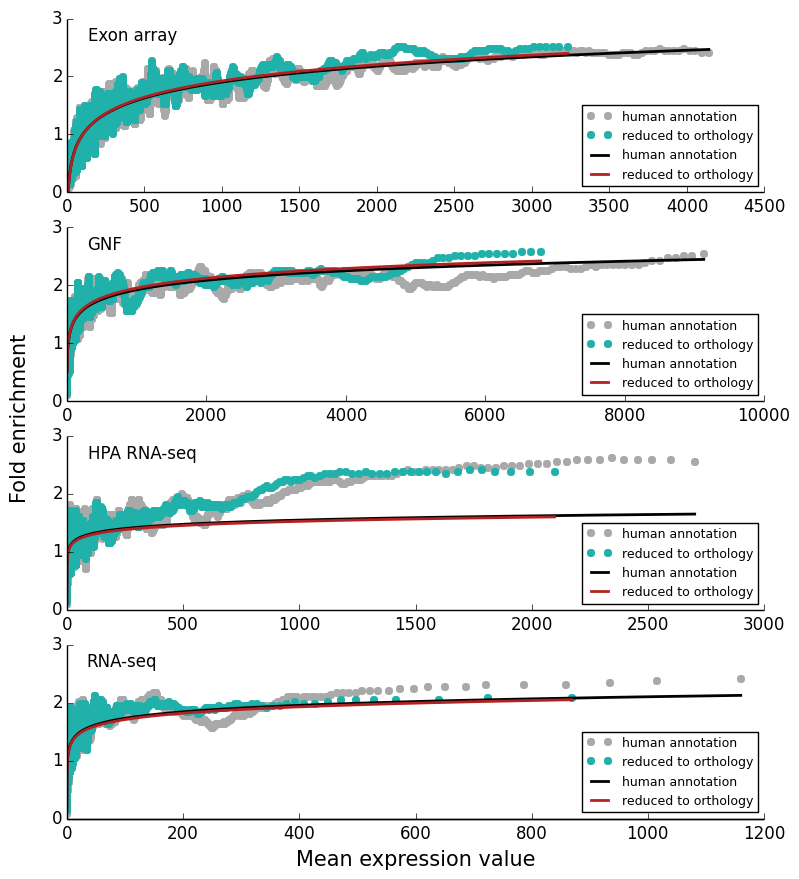


*Supplementary Figure S2. Comparison between benchmarking the human datasets using the full human UniProtKB tissue annotation dataset as gold standard (gray) vs. using a smaller gold standard dataset, obtained by selecting genes with a 1:1 ortholog in rat (green). There are only minor changes in fold enrichment values and by fitting the relationship between mean expression values and fold enrichments in the two scenarios we obtain almost identical curves, indicating that the scores are robust to reducing the size of the gold standard dataset. We chose to display results for rat orthology, because the smaller the dataset, the more problematic it could be, and rat, among the three organisms, has the smallest orthology-based gold standard set.*


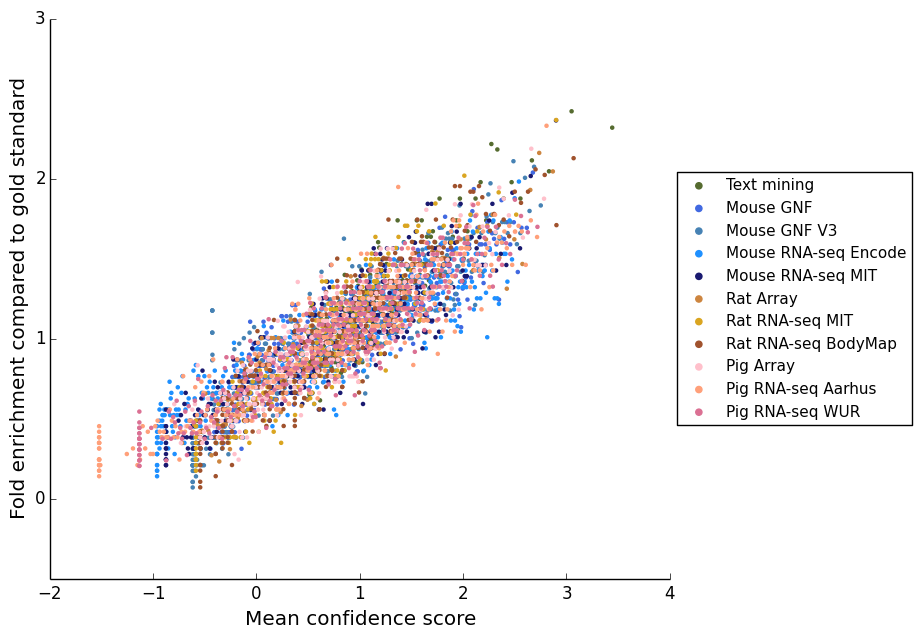


*Supplementary Figure S3. Fold enrichment scores, quantifying the quality of all gene-tissue associations from all datasets, are transformed to star confidence scores by calibration to the text mining results for human gene-tissue associations. The figure shows that after score calibration, the same confidence score corresponds to the same quality.*

*Supplementary Figure S4. Pearson's correlation coefficients between "star" confidence scores for genes being expressed in heart, kidney, liver and nervous system. Only genes common between all datasets are considered (1:1:1:1 orthologous genes with a positive star score in at least one tissue in each dataset). A total of only 1528 genes satisfy this constraint, and the rat array dataset was left out from this analysis due to reducing the number of common genes even further. This analysis shows patterns of correlation between consistent with those observed when performing the analyses for tissues and genes common between each two datasets, with RNA-seq datasets generally having a stronger correlation with other datasets.*

*Supplementary Figure S5. Pearson's correlation coefficients between "star" confidence scores across tissues and datasets, for six of the tissues covered by most of the datasets. Common genes between each two datasets are considered. We observe a positive correlation coefficient between expression in the same tissue across experiments, technologies and organisms, while the correlation between expressions across different tissues is negative and equally low when different tissues are part of the same dataset.*
